# Supplementary material for: Force generation by a propagating wave of supramolecular nanofibers
Source: Nat Commun. 2020 Jul 15;11:3541. doi: 10.1038/s41467-020-17394-z (PMC7363860; doi:10.1038/s41467-020-17394-z)
Supplement: Supplementary file 1 — Supplementary information [file 41467_2020_17394_MOESM1_ESM.pdf]

Supplementary Information

Force generation by a propagating wave of supramolecular nanofibers

Kubota et al.

Supplementary Methods

General. Unless stated otherwise, all commercial reagents were used as received. Thin layer chromatography (TLC) was performed on silica gel $60F_{254}$ (Merck). ^{1}H NMR spectra were obtained on a Varian Mercury 400 spectrometer with residual non-deuterated solvents (CD_3OD: 3.31 ppm for ^{1}H) as the internal reference. ESI mass spectra were recorded using an Exactive (Thermo Scientific). Reversed-phase HPLC (RP-HPLC) was carried out on a Hitachi Chromaster system equipped with a diode array and YMC-Pack Triart C18 or ODS-A columns. All runs used linear gradients of acetonitrile (ACN) containing 0.1% trifluoroacetic acid (TFA) and 0.1% aqueous TFA. The images of confocal laser scanning microscopy (CLSM) were acquired by a FV1000 (Olympus) and a LSM800 (Carl Zeiss Microscopy). UPlanSApo 100× (1.40 numerical aperture, oil immersion, Olympus), UPlanSApo 4× (0.16 numerical aperture, Olympus), Plan-Apochromat 20× (0.8 numerical aperture, Carl Zeiss), and αPlan-Apochromat 100× (1.46 numerical aperture, Carl Zeiss) were used. The fluorescent intensity of the CLSM images was calculated by Fiji.[1]

Preparation of a homogeneous BPmoc-F_3 solution. A BPmoc-F_3 powder was suspended in an aqueous buffer (50 mM HEPES, pH 7.4), and heated until dissolving. After cooling to room temperature, 10× stock solutions of BP-TMR (3.4 μM in 50 mM HEPES, pH 7.4) and/or GOx (10 mg/mL in 50 mM HEPES, pH 7.4 containing 5% (v/v) glycerol) were added.

Zn^{2+} ion-induced gelation of BPmoc-F_3. To a freshly-prepared aqueous solution of BPmoc-F_3 (2.4 mM, 0.15 wt%, 50 mM HEPES buffer (pH 7.4), 100 μL) was added a 10× stock solution of $Zn(NO_3)_2$ (12 mM in H_2O, 10 μL) or H_2O (10 μL). The resulting solution was allowed to stand for 30 min at room temperature. After confirming gelation, a 10× stock solution of EDTA (12 mM, 50 mM HEPES, pH 7.4, 10 μL) or buffer (10 μL) was added to the hydrogel, and incubated for 30 min at room temperature. The sample state (gel or sol) was confirmed by the tube inversion method. Photos were taken by iPhone 5 (Apple Inc.).

Glucose response of GOx-encapsulated Zn^{2+}-induced hydrogels. To a freshly-prepared aqueous solution of BPmoc-F_3 and GOx (2.4 mM and 1 mg/mL, respectively, 50 mM HEPES containing 0.5% glycerol, pH 7.4, 110 μL) was added a 10× stock solution of $Zn(NO_3)_2$ (12 mM, 0.5 eq, 10 μL in H_2O). The resulting solution was incubated for 30 min at room temperature to form a transparent hydrogel. To the resultant hydrogel was added a 10× stock solution of glucose (48 mM, 2.0 eq, 10 μL in H_2O), and then the resulting hydrogel was incubated at 30 ºC for 2 h. The state (gel or sol) was determined by the tube inversion method. To determine the reaction rate by RP-HPLC, a solution of *p*-nitrobenzenesulfonamide (1.6

mM, 100 μL in CH_3CN) was added to the gel/sol as an internal standard, and the resultant solution was analyzed by RP-HPLC.

Real-time imaging of Zn^{2+}-induced nanofiber formation. A freshly-prepared aqueous solution of BPmoc-F_3 and BP-TMR (1.6 mM and 0.32 μM, respectively, 50 mM HEPES, pH 7.4, 10 μL) was deposited on a Matsunami glass bottom dish (non-coated, thickness: 0.16~0.19 mm, catalog number: D11530H). A 10× stock solution of $Zn(NO_3)_2$ (8 mM, 1.0 μL in H_2O) or H_2O (1.0 μL) was added to the solution 1 min after starting time-lapse CLSM imaging. In supplementary Fig. 8, a 10× stock solution of glucose (32 mM, 1 μL), GOx (10 mg/mL, 1 μL), or H_2O (1 μL) was added 30 min before $Zn(NO_3)_2$ addition. The images and time-lapse movies of the Zn^{2+}-induced formation of supramolecular nanofibers were obtained by LSM800 with the 100× objective. The overall fluorescent intensity of the field of view was calculated by Fiji.

Real-time imaging of glucose-responsive fiber degradation. A freshly-prepared aqueous solution of BPmoc-F_3, BP-TMR, and GOx (1.6 mM, 0.32 μM and 1.0 mg/mL, respectively, 50 mM HEPES containing 0.5% glycerol, pH 7.4, 11 μL) was deposited on a Matsunami glass bottom dish. To this solution was added a 10× stock solution of $Zn(NO_3)_2$ (8 mM, 1.0 μL in H_2O). The resulting solution was incubated for 30 min at room temperature. A 10× stock solution of glucose (32 mM, 2.0 eq, 1.0 μL in H_2O) or H_2O was added one minute after starting the time-lapse imaging. The time-lapse movies of nanofiber degradation were obtained by FV1000 with the 100× objective. The overall fluorescent intensity of the field of view was calculated by Fiji.

HPLC analysis of degradation kinetics of BPmoc-F_3. To a freshly-prepared aqueous solution of BPmoc-F_3 and GOx (0.2 mM and 1 mg/mL, respectively, 50 mM HEPES containing 0.5% glycerol, pH 7.4, 110 μL) was added a solution of $Zn(NO_3)_2$ (8 mM, 10 μL) or H_2O (10 μL). To the resultant mixture was added a solution of glucose (32 mM, 10 μL), and then the resulting solution was incubated at 20 ºC for 15, 30, and 60 min. To determine the reaction rate by RP-HPLC, a solution of *p*-nitrobenzenesulfonamide (0.32 mM, 100 μL in CH_3CN) was added to the reaction solution as an internal standard. The resultant solution was immediately analyzed by RP-HPLC.

Time-lapse CLSM imaging of the propagating wave. A freshly-prepared aqueous solution of BPmoc-F_3, BP-TMR, and GOx (1.6 mM, 0.32 μM, and 1 mg/mL, respectively, 50 mM HEPES containing 0.5% glycerol, pH 7.4, 5.5 μL) was deposited between a Matsunami micro cover glasses (bottom: non-coat, 30×40 mm, 0.12–0.17 mm, top: 18×18 mm, 0.12–0.17 mm, non-coat. The cover glasses were sticked with four double-faced adhesive tape

(Nichiban). A mixed solution of $Zn(NO_3)_2$ and glucose (4 mM and 16 mM, respectively, 1.0 μL in H_2O) was added at the right edge of the droplet one minute after time-lapse imaging was started. The volumes of the droplet containing BPmoc-F_3/BP-TMR/GOx and the solution of $Zn(NO_3)_2$/glucose were kept constant when applying different concentration of GOx and glucose. The time-lapse movies of a propagating wave were obtained by FV1000. In the case of CLSM imaging with the 100× objective, the images were acquired at ~1 mm from the right edge of the droplet. In the case of the time-lapse movie acquired with the 100× objective, the fluorescent intensity of the ROI [size (x × y): 10 pixels × 512 pixels] at desired x coordinates was calculated by Fiji. When using the 4× objective, fluorescent intensity of the ROI [size (x × y): 10 pixels × 100 pixels] at desired x coordinates was calculated by Fiji.

CLSM imaging upon treatment of $Zn(NO_3)_2$ followed by glucose. The preparation of the droplet was the same as "time-lapse CLSM imaging of the propagating wave". A solution of $Zn(NO_3)_2$ (8 mM, 0.5 μL in H_2O) was added to the right edge of the droplet. After 30 min, a solution of glucose (32 mM, 0.5 μL in H_2O) was added to the right edge of the droplet one minute after starting time-lapse CLSM imaging. The images were acquired at ~1 mm from the right edge of the droplet by FV1000.

CLSM imaging upon treatment of glucose followed by $Zn(NO_3)_2$. The preparation of the droplet was the same as "time-lapse CLSM imaging of the propagating wave". A solution of glucose (32 mM, 0.5 μL in H_2O) was added to the right edge of the droplet. After 5 min, a solution of $Zn(NO_3)_2$ (8 mM, 0.5 μL in H_2O) was added to the right edge of the droplet one minute after starting time-lapse CLSM imaging. The images were acquired at ~1 mm from the right edge of the droplet by FV1000.

Preparation of Oregon Green-modified polystyrene beads. To a suspension of polystyrene beads (micromod, product code 01-01-502, diameter 500 nm, NH_2-modified, 50 mg/mL, 100 μL) were added 100 mM HEPES buffer (pH 8.0, 100 μL), PEG300-NHS (3.3 mg, 10 μmol, Quanta Biodesign), and Oregon Green 488-NHS (0.3 μmol, 6 μL in DMSO, Thermo Fisher). The mixture was incubated at room temperature for 24 h. The resultant mixture was centrifuged (13500 rpm, 4 ºC, 5 min), the supernatant was removed, and then H_2O (1 mL) was added. This washing step was repeated 3 times. The washed beads were collected by centrifuge (13500 rpm, 4 ºC, 5 min). To the resulting beads, H_2O (100 μL) was added to obtain a suspension of Oregon Green-modified PEG-coated polystyrene beads.

***In situ* CLSM imaging of the bead displacement.** The droplet containing BPmoc-F_3, BP-TMR, GOx, and Oregon Green-modified beads (1.6 mM, 0.32 μM, 1 mg/mL, and 20 μg/mL, respectively, 5.5 μL in 50 mM HEPES, pH 7.4) were prepared by the same protocol as "time-

lapse CLSM imaging of the propagating wave." A mixture of $Zn(NO_3)_2$ and glucose (4 mM and 16 mM, respectively, 1.0 μL in H_2O) was added to the droplet. The images were acquired by LSM800 with a 20× objective lens.

According to Stokes's law at low Reynold number, the force generated by the propagating wave was determined by the following equation.

$$F_d = 6\pi\mu R v, \text{(1)}$$

Here, F_d, μ, R, and v are defined as the force applied to a spherical particle, viscosity of the solution, a radius and velocity of a spherical particle (0.25 μm and 0.4 μm/s), respectively. We determined viscosity of a buffer (50 mM HEPES, pH 7.4), a solution of BPmoc-F_3 before and after $Zn(NO_3)_2$ to be 1.1, 1.3, 2.5 mPa·s, respectively. Although the exact viscosity of the solution during the propagating wave could not be determined, we assumed that the maximum viscosity was lower than 2.5 mPa·s because the fluorescent intensity of the nanofibers in the propagating wave was lower than that in the mixture of BPmoc-F_3, BP-TMR, and $Zn(NO_3)_2$. Therefore, the force generated by the propagating wave was calculated to be the order of 0.005 pN with the viscosity value of 2.5 mPa·s.

Determination of the persistence length. To a solution of BPmoc-F_3 and BP-TMR (1.2 mM, 0.34 μM, 50 mM HEPES (pH 7.4), 50 μL), a solution of $Zn(NO_3)_2$ (8 mM, 5.0 μL in H_2O) was added. After incubation at room temperature for 30 min, the resultant viscous solution was vortexed for 30 sec and transferred on a glass bottom dish. Time-lapse CLSM images were acquired at 33 frames/sec by LSM800 with a 100× objective lens. Binary images were skeletonized and further spline interpolated using MATLAB to obtain sub-pixel fiber contour coordinates. The coordinates were used to calculate the 2-dimensional persistence length L_p (supplementary Fig. 19a):

$$\langle\cos[\Delta\theta(s)]\rangle = \exp\left(-s/2L_\mathrm{p}\right), \text{(2)}$$

where $\Delta\theta(s)$ is the angle change over the arc length s.

Organic synthesis. BPmoc-F_3 and compound **1** were synthesized according to our previous literatures[2,3].

BP-TMR

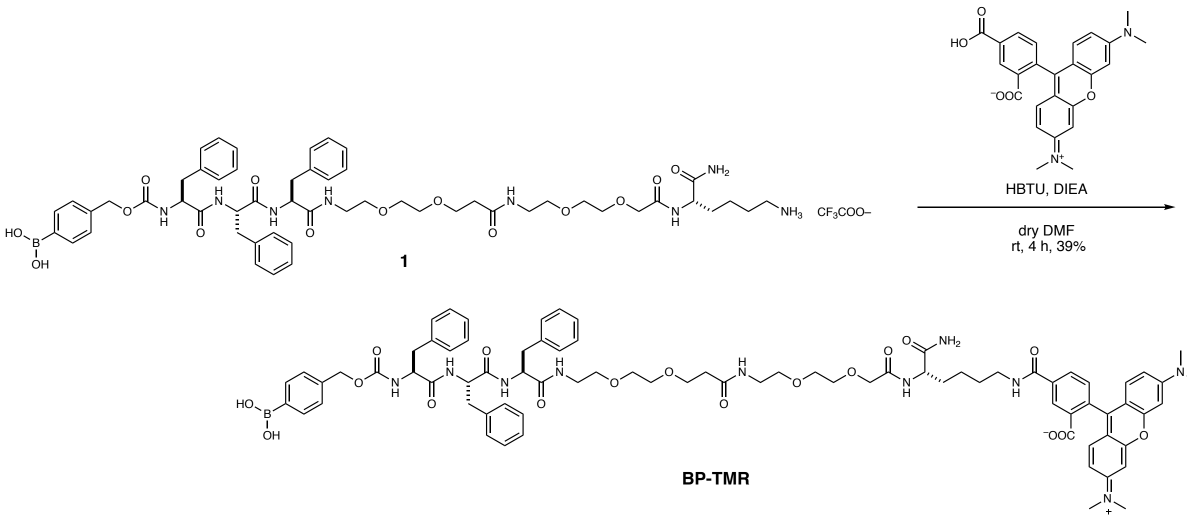

To a solution of dry DMF (1 mL) and 5-carboxytetramethylrhodamine (8.4 mg, 19 μmol, 1.0 eq) were added HBTU (8.7 mg, 23 μmol, 1.2 eq), DIEA (10 μL, 57 μmol, 3.0 eq) and **1** (25 mg, 23 μmol, 1.2 eq) under N_2 atmosphere. The mixture was stirred at rt for 4 h and the reaction was monitored by TLC (silica, $CHCl_3$: MeOH = 3:1 with a small amount of AcOH). The mixture was evaporated to remove the solvent. The crude product was purified by RP-HPLC (gradient: CH_3CN (0.1% TFA) : H_2O (0.1% TFA) = 35/65 (0 min) to 65/35 (35 min), column: YMC triart C-18, the target was eluted at 21.2 min). The target fractions were lyophilized to obtain BP-TMR (11 mg, 7.4 μmol, 39%) as a red solid.

^{1}H NMR (400 MHz, CD_3OD, rt): δ 1.44–1.56 (m, 2H), 1.70–1.82 (m, 3H), 1.89–2.00 (m, 1H), 2.44–2.47 (t, *J* = 6.4 Hz, 2H), 2.68–3.13 (m, 6H), 3.28–3.30 (m, a solvent peak was overlapped), 3.34–3.71 (m, 20H), 4.02 (s, 2H), 4.28–4.31 (dd, *J* = 5.4, 9.4 Hz, 1H), 4.46–4.57 (m, 3H), 4.91–5.00 (d, *J* = 16.4 Hz, 2H), 6.96–7.25 (m, 25H), 7.47–7.49 (d, *J* = 8.0 Hz, 1H), 8.24–8.26 (d, *J* = 8.0 Hz, 1H), 8.76 (s, 1H).

HR-FTMS (ESI): calcd for $[M+H]^+$: *m/z* = 1481.6835; found: 1481.6806.

Supplementary Discussion

Numerical analysis of the propagating wave of supramolecular nanofibers. A model was developed based on a reaction-diffusion mechanism. We defined variables as shown below.

$[n]$: Concentration of the nanofiber

$[m]$: Concentration of the monomer

$[x]$: Concentration of the formation stimulus

$[y]$: Concentration of the degradation stimulus

D_n, D_m, D_x, D_y: diffusion coefficient of each component

where we assumed $D_n << D_m \leq D_x \leqq D_y$ according to molecular sizes.

For nanofiber formation reactions from the monomer, we supposed three basic reactions:

$k_1[m]^\alpha[x]$: Nanofiber formation between the monomers assisted by the formation stimulus x

$k_5[n]^\beta[m][x]$: Nanofiber elongation assisted by the formation stimulus x

$k_2[n][m]$: Nanofiber elongation

For the degradation reactions of the monomer and nanofiber, we set two basic reactions:

$k_3[n][y]$: Nanofiber degradation by the degradation stimulus y

$k_4[m][y]$: Monomer degradation by the degradation stimulus y

Considering all the basic reactions, we obtained a set of reaction-diffusion equations as shown below:

$$\frac{\partial n}{\partial t} = D_n \nabla^2 n + k_1 m^\alpha x + k_2 nm - k_3 ny + k_5 n^\beta mx, \quad (3)$$

$$\frac{\partial m}{\partial t} = D_m \nabla^2 m - k_1 m^\alpha x - k_2 nm - k_4 my, \quad (4)$$

$$\frac{\partial x}{\partial t} = D_x \nabla^2 x, \quad (5)$$

$$\frac{\partial y}{\partial t} = D_y \nabla^2 y, \quad (6)$$

In a numerical simulation, we assumed that α and β are equal to one as linear approximation because the monomer concentration was limited under the initial concentration and the relationship between the fluorescent intensity, the fiber length, and reaction dynamics remains unknown.

In a numerical analysis, we omitted time dependence changes of the monomer and the formation stimulus because the concentration of the monomer is spatially homogeneous especially for a front region of the propagating wave.

$$\frac{\partial n}{\partial t} = D_n \nabla^2 n + k_1 m^\alpha x + k_2 nm + k_5 n^\beta mx - k_3 ny, (7)$$

$$\frac{\partial y}{\partial t} = D_y \nabla^2 y, (8)$$

From these equations, the wave velocity c could be calculated to be $c \sim \tau D_y/t$, which is inversely proportional to time. The normalization unit τ is defined below. In detail, dimensionless equations of the above mentioned ones are,

$$\varepsilon\tau \frac{\partial u}{\partial t} = \varepsilon^2 \nabla^2 u + f(n, m, x) - uv, (9)$$

$$\frac{\partial v}{\partial t} = D_v \nabla^2 v, (10)$$

where $\varepsilon\tau$ is a normalization coefficient, D_n is replaced by ε^2 (note, $\varepsilon << 1$ is postulated from $D_n << D_y$), $k_1 m^\alpha x + k_2 nm + k_5 n^\beta mx$ and $k_3 ny$ are replaced with a nonlinear term $f(n, m, x)$ and uv, respectively. These equations were analyzed along the same way done in a reaction-diffusion system.[4] The direction of the propagating wave was limited in one dimension, and the object frame of the wave front z is set. The stationary solution $u(z)$ obeys,

$$-c\varepsilon\tau \frac{du}{dz} = \varepsilon^2 \frac{d^2 u}{dz^2} + f(u, m, x) - v_I, (11)$$

where v_I is the value of uv at the wave front. Both the degradation term v_I and the reaction term f should affect stationary at the propagating wave front $z = 0$. Independently diffusing v can be approximately evaluated around $z = 0$ as,

$$v_I \sim v_0 (1 - \frac{c^2 t}{4D}), (12)$$

From equation (9), $\frac{c\tau}{\sqrt{(c\tau)^2+4}} = 1 - 2f - 2v_I$ was yielded[4], and then,

$$\frac{c\tau}{\sqrt{(c\tau)^2+4}} - \frac{c^2 t}{2D} = const., (13)$$

where $const. = 1 - 2f - 2v_0$. Applying series expansion to the denominator of the first term,

$$-\frac{\tau^3}{16} c^3 + \frac{c}{2}\left(\tau - \frac{ct}{D}\right) = const., (14)$$

is yielded. Considering the term ct corresponds to the displacement of the wave front, positive valued c solution requires at least,

$$c < \frac{\tau D}{t}, (15)$$

The upper limit or the order of magnitude of the velocity is determined by the condition. According to the analysis, the velocity is proportional to the formation kinetics of nanofibers

and the diffusion coefficient of the degradation stimulus, and inversely proportional to time under the present experimental setup.

Supplementary Figures

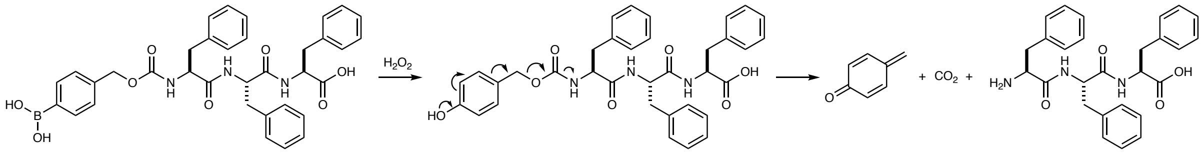

Supplementary Fig. 1 | Reaction mechanism of BPmoc-F_3 degradation by H_2O_2.[2]

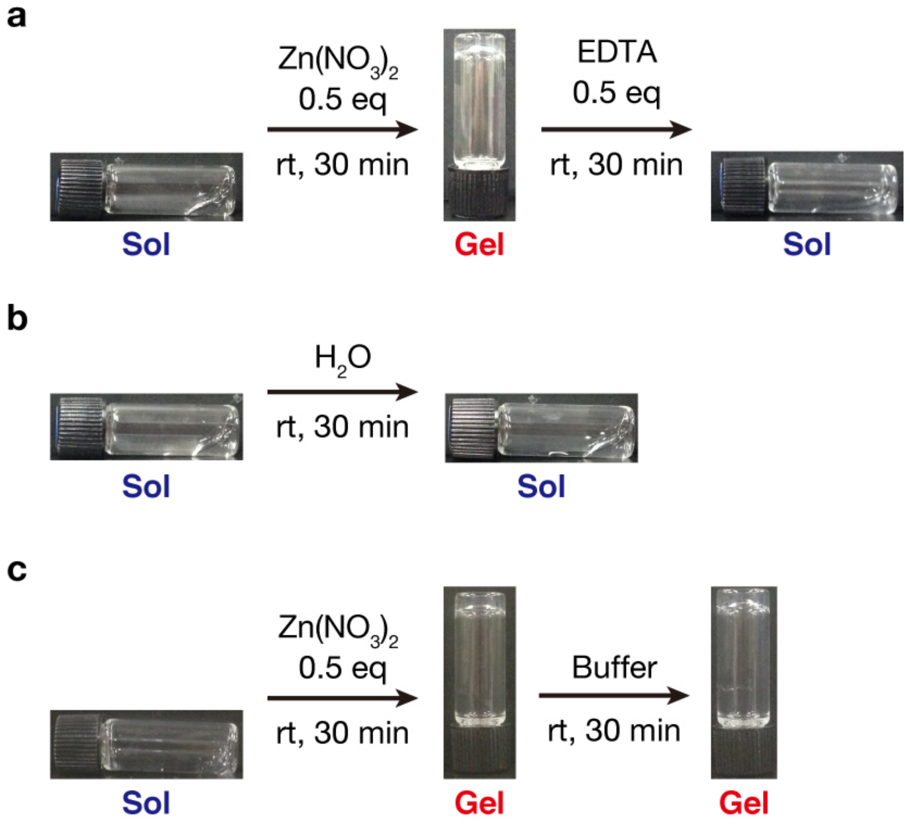

Supplementary Fig. 2 | Zn^{2+}-induced hydrogelation of BPmoc-F_3. (**a**) Sol-gel and gel-sol transition upon treatment of $Zn(NO_3)_2$ and EDTA, respectively. Neither (**b**) Sol-gel nor (**c**) gel-sol transition took place by addition of H_2O or buffer instead of $Zn(NO_3)_2$ or EDTA, respectively. Condition: [BPmoc-F_3] = 2.4 mM, [$Zn(NO_3)_2$] = 1.2 mM, [EDTA] = 1.2 mM, 50 mM HEPES, pH 7.4.

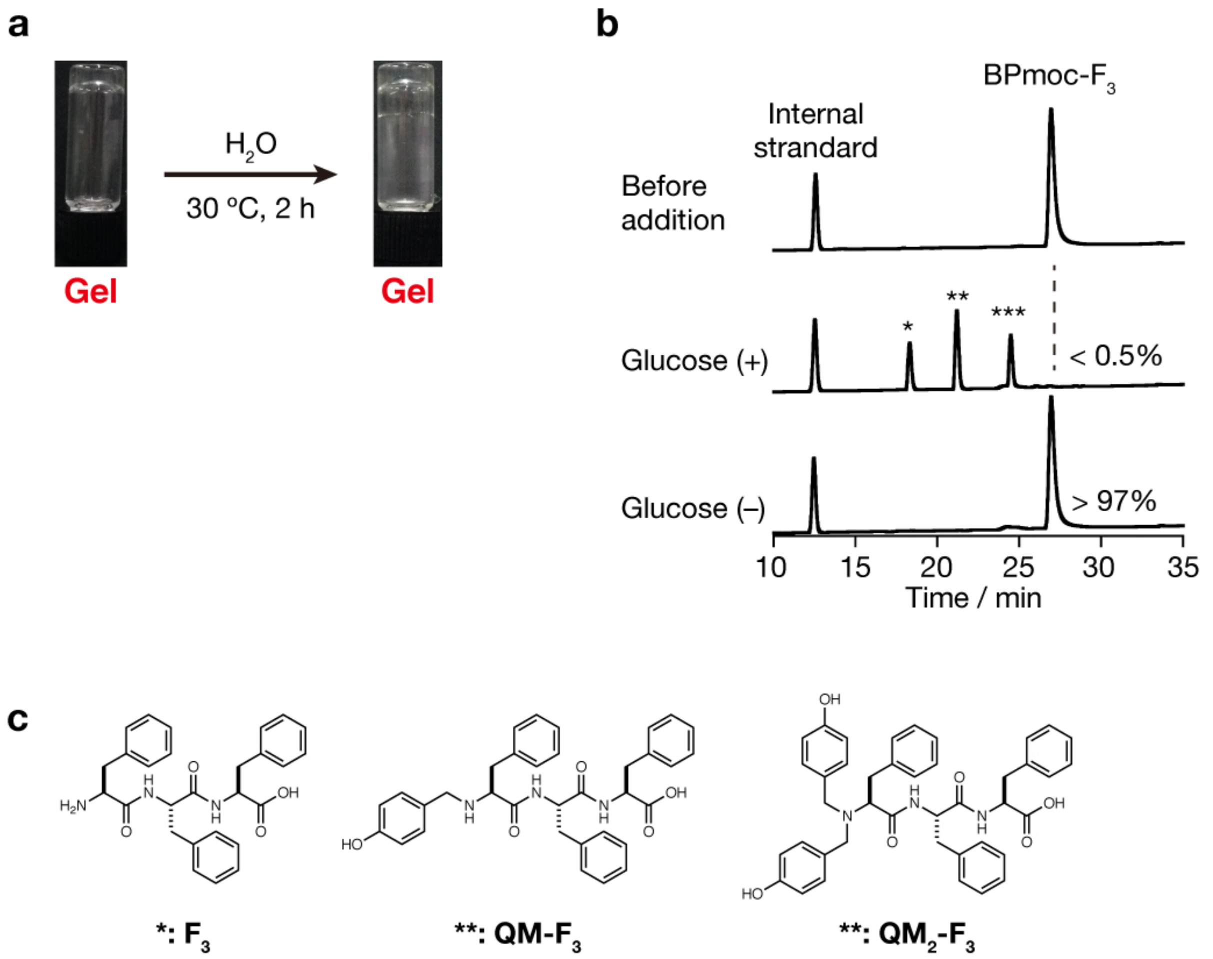

Supplementary Fig. 3 | Glucose response of the Zn^{2+}-induced BPmoc-F_3 hydrogel containing GOx. (**a**) Gel-sol transition did not proceed by addition of H_2O. (**b**) HPLC analysis of the Zn^{2+}-induced BPmoc-F_3 hydrogel (upper) before and after addition of (middle) glucose or (bottom) H_2O. Internal standard: *p*-nitrobenzenesulfonamide. (**c**) Plausible structures of degradation products.[2] Condition: [BPmoc-F_3] = 2.4 mM, [GOx] = 1 mg/mL, [$Zn(NO_3)_2$] = 1.2 mM, [glucose] = 4.8 mM, 50 mM HEPES, pH 7.4.

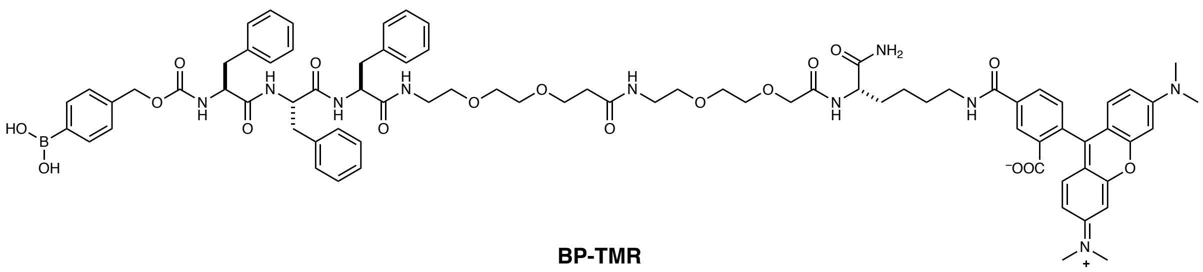

BP-TMR

Supplementary Fig. 4 | Chemical structure of a fluorescent probe, BP-TMR.

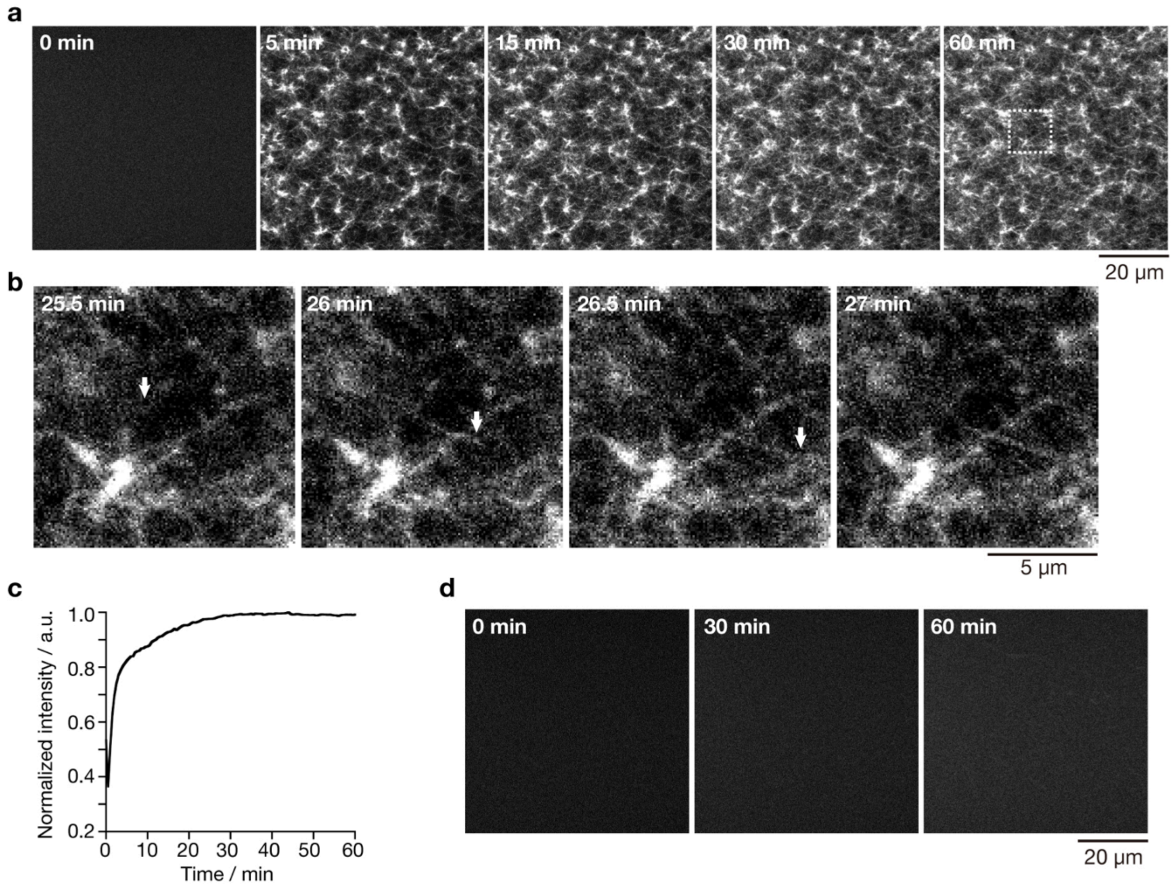

Supplementary Fig. 5 | Zn^{2+}-induced nanofiber formation of BPmoc-F_3. (**a**) Time-lapse CLSM imaging of BPmoc-F_3 nanofiber formation upon treatment of $Zn(NO_3)_2$. (**b**) Magnified view of the nanofiber elongation process. The elongation velocity was estimated to be 6.4 μm/min. (**c**) Time profile of total fluorescent intensity of the field of view. (**d**) No nanofiber formation was observed by addition of H_2O instead of $Zn(NO_3)_2$. Condition: [BPmoc-F_3] = 1.6 mM, [BP-TMR] = 0.34 μM, [$Zn(NO_3)_2$] = 0.8 mM, 50 mM HEPES, pH 7.4, 30 ºC.

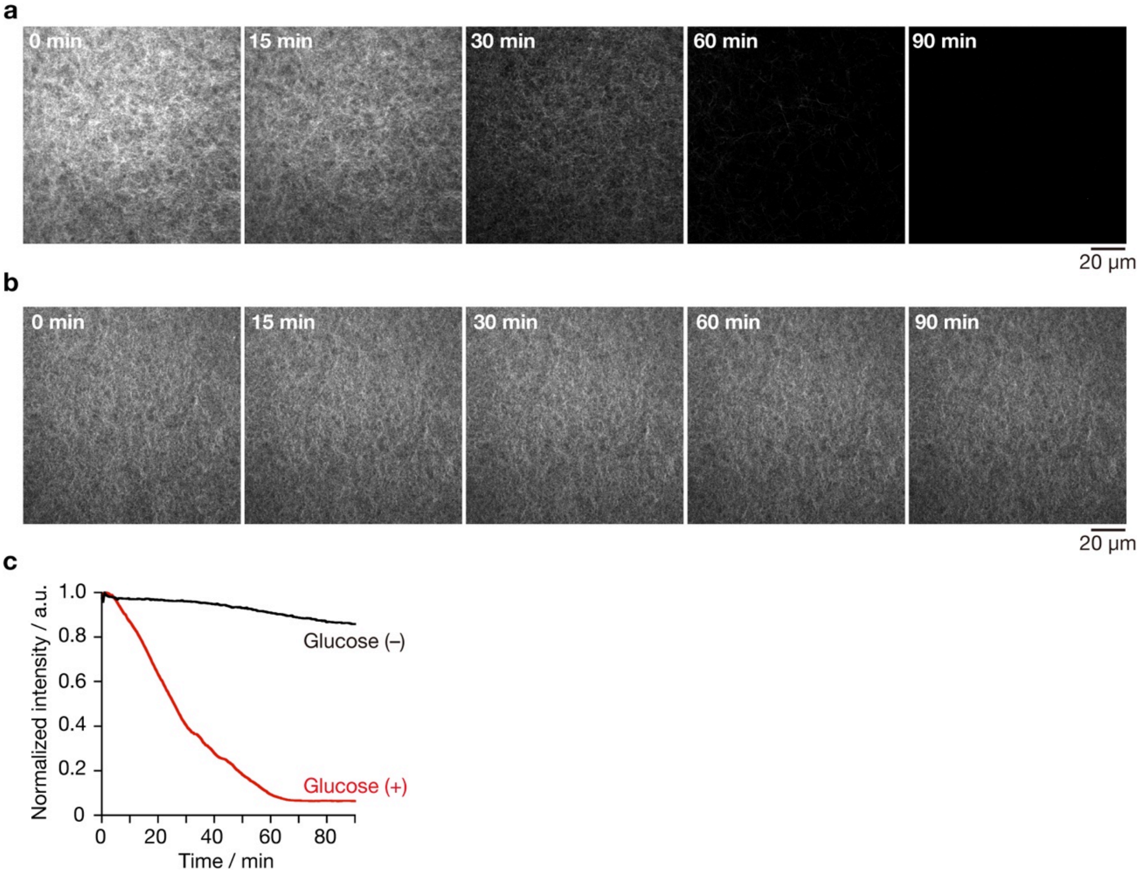

Supplementary Fig. 6 | Glucose response of the Zn^{2+}-induced BPmoc-F_3 nanofibers. Time-lapse CLSM imaging of degradation of the Zn^{2+}-induced BPmoc-F_3 nanofibers upon treatment of (**a**) glucose and (**b**) H_2O. (**c**) Time profile of total fluorescent intensity of the field of view. The decrease of the fluorescent intensity in supplementary Fig. 6b was due to photobleaching of the fluorescent probe (BP-TMR). As shown in supplementary Fig. 7, BP-TMR showed a negligible effect on the nanofiber degradation. Condition: [BPmoc-F_3] = 1.6 mM, [BP-TMR] = 0.34 µM, [GOx] = 1 mg/mL, [$Zn(NO_3)_2$] = 0.8 mM, [glucose] = 0 or 3.2 mM, 50 mM HEPES, pH 7.4, 30 ºC.

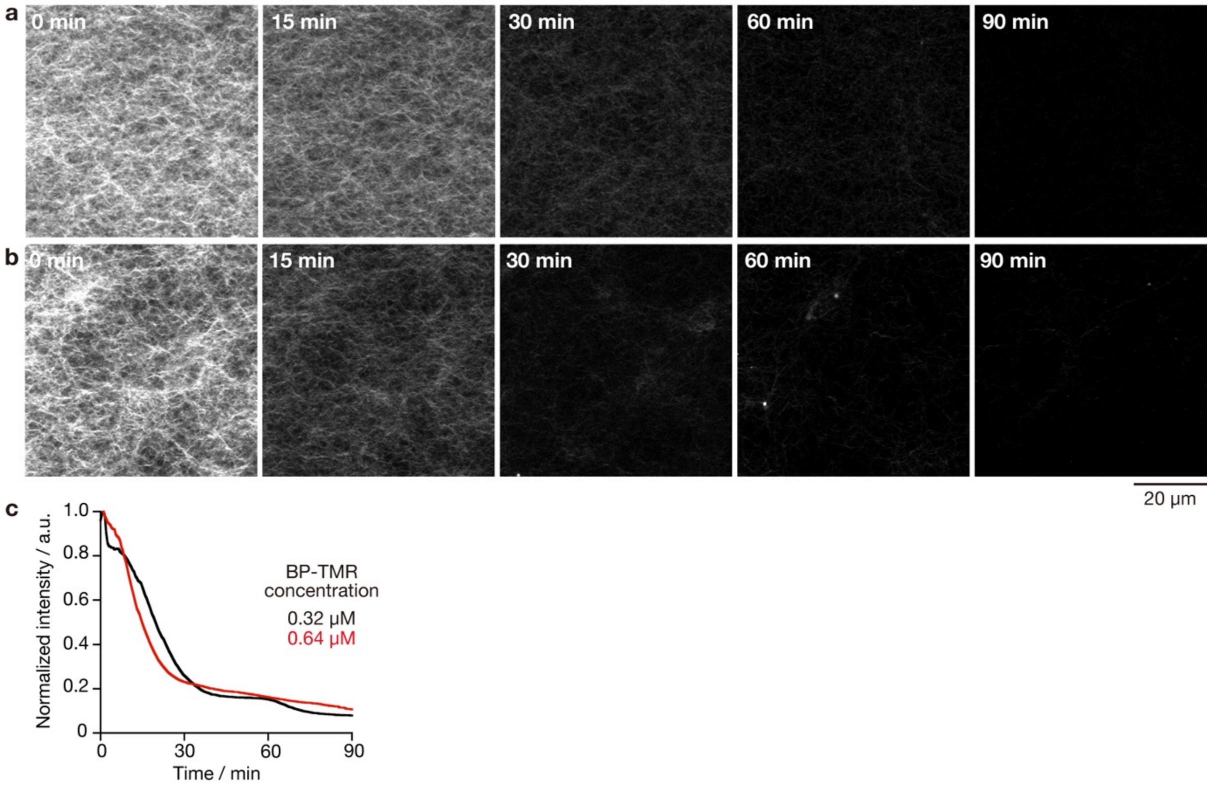

Supplementary Fig. 7 | Concentration dependence of BP-TMR on degradation of Zn^{2+}-induced BPmoc-F_3 nanofibers. Time-lapse CLSM imaging of degradation of the Zn^{2+}-induced BPmoc-F_3 nanofibers in the presence of (**a**) 0.32 μM and (**b**) 0.64 μM of BP-TMR. (**c**) Time profile of total fluorescent intensity of the field of view. Condition: [BPmoc-F_3] = 1.6 mM, [BP-TMR] = 0.34 or 0.64 μM, [GOx] = 1 mg/mL, [$Zn(NO_3)_2$] = 0.8 mM, [glucose] = 3.2 mM, 50 mM HEPES, pH 7.4, 30 ºC.

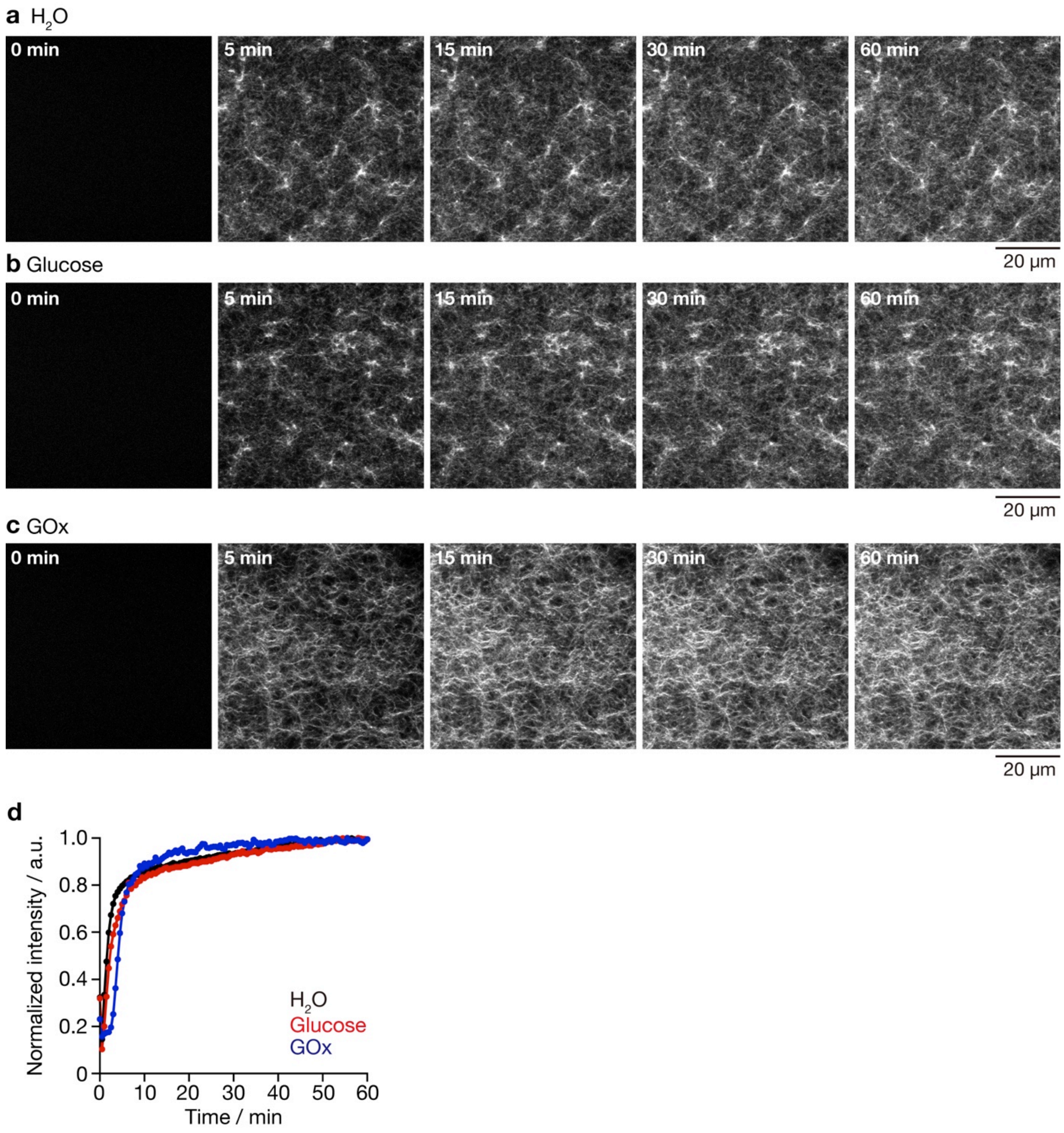

Supplementary Fig. 8 | Glucose and GOx did not affect the formation kinetics of Zn^{2+}-induced BPmoc-F_3 nanofibers. Time-lapse CLSM imaging of formation of the Zn^{2+}-induced BPmoc-F_3 nanofibers in the presence of (**a**) H_2O, (**b**) glucose, and (**c**) GOx. (**d**) Time profile of total fluorescent intensity of the field of view. Condition: [BPmoc-F_3] = 1.6 mM, [BP-TMR] = 0.34 μM, [$Zn(NO_3)_2$] = 0.8 mM, [glucose] = 0 or 3.2 mM, [GOx] = 0 or 1 mg/mL, 50 mM HEPES, pH 7.4, 30 ºC.

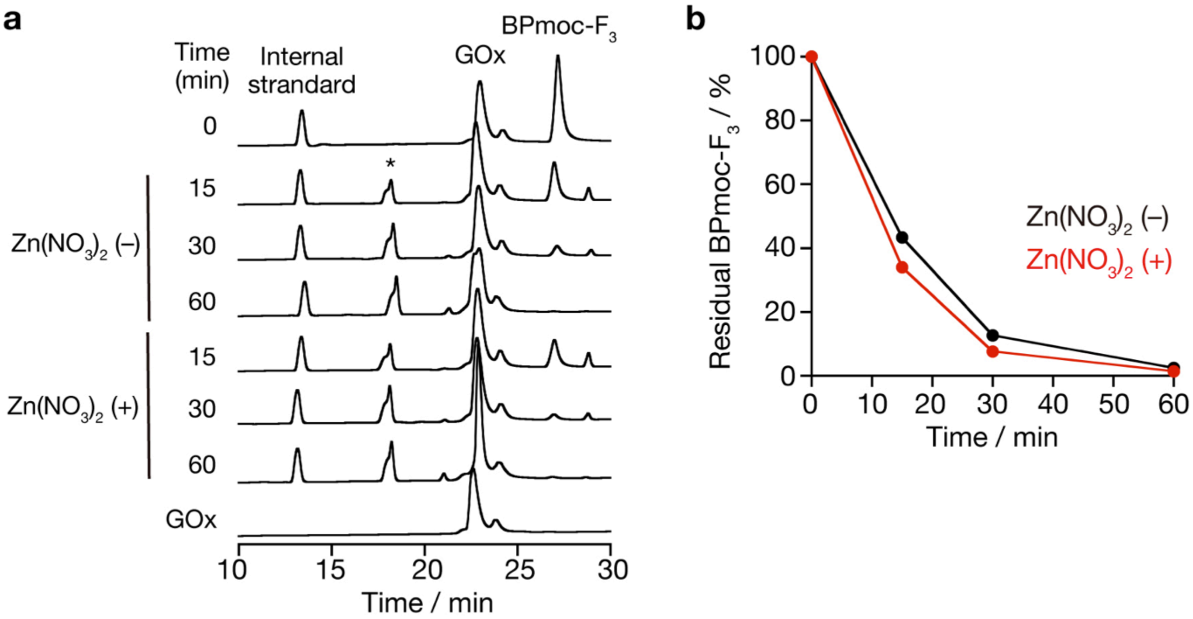

Supplementary Fig. 9 | $Zn(NO_3)_2$ did not affect the degradation kinetics of BPmoc-F_3. (**a**) HPLC analysis of BPmoc-F_3 degradation by GOx/glucose in the absence and presence of $Zn(NO_3)_2$. Concentrations of $Zn(NO_3)_2$, glucose, and GOx were the same as that in the propagating wave. (**b**) Plot of the residual BPmoc-F_3 amount. Condition: [BPmoc-F_3] = 0.2 mM, [$Zn(NO_3)_2$] = 0 or 0.8 mM, [glucose] = 3.2 mM, [GOx] = 1 mg/mL, 50 mM HEPES, pH 7.4, 20 ºC.

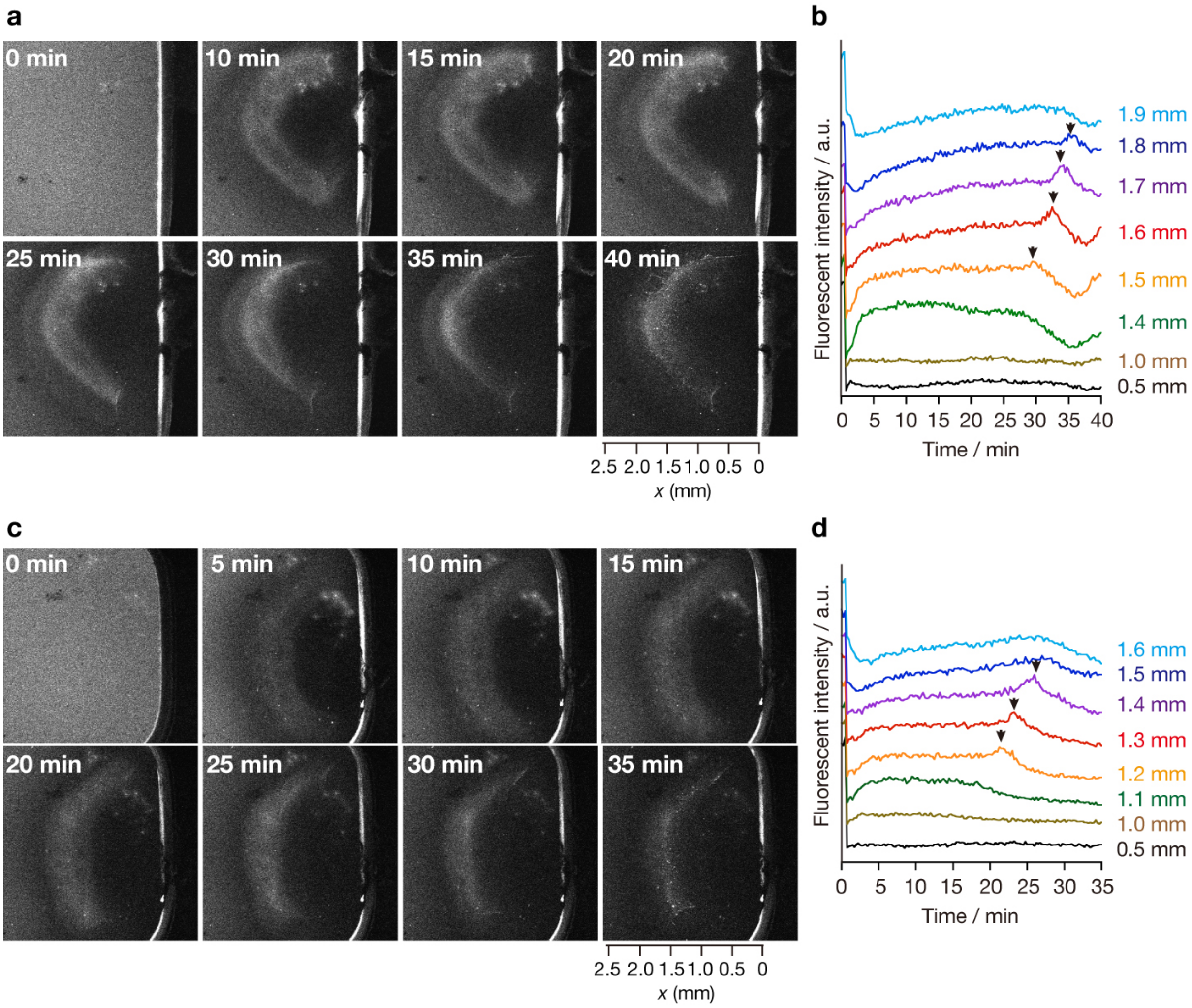

Entry	Distance (μm)	Velocity (μm/min)
1	354	61.6
2	373	59.6
3	298	47.7
Average	340 ± 40	54 ± 8

Supplementary Fig. 10 | The average distance and velocity of the propagating wave are reproducible. (**a**, **c**) Time-lapse CLSM imaging of the propagating wave of supramolecular nanofibers. (**b**, **d**) Time course of fluorescent intensity changes at distinct *x*-coordinates. (**e**) Table of traveling distances and velocity of the propagating wave. The data represent the average ± standard deviation. Condition: [BPmoc-F_3] = 1.6 mM, [BP-TMR] = 0.34 μM, [GOx] = 1 mg/mL, [$Zn(NO_3)_2$] = 0.8 mM, [glucose] = 3.2 mM, 50 mM HEPES, pH 7.4, 30 ºC.

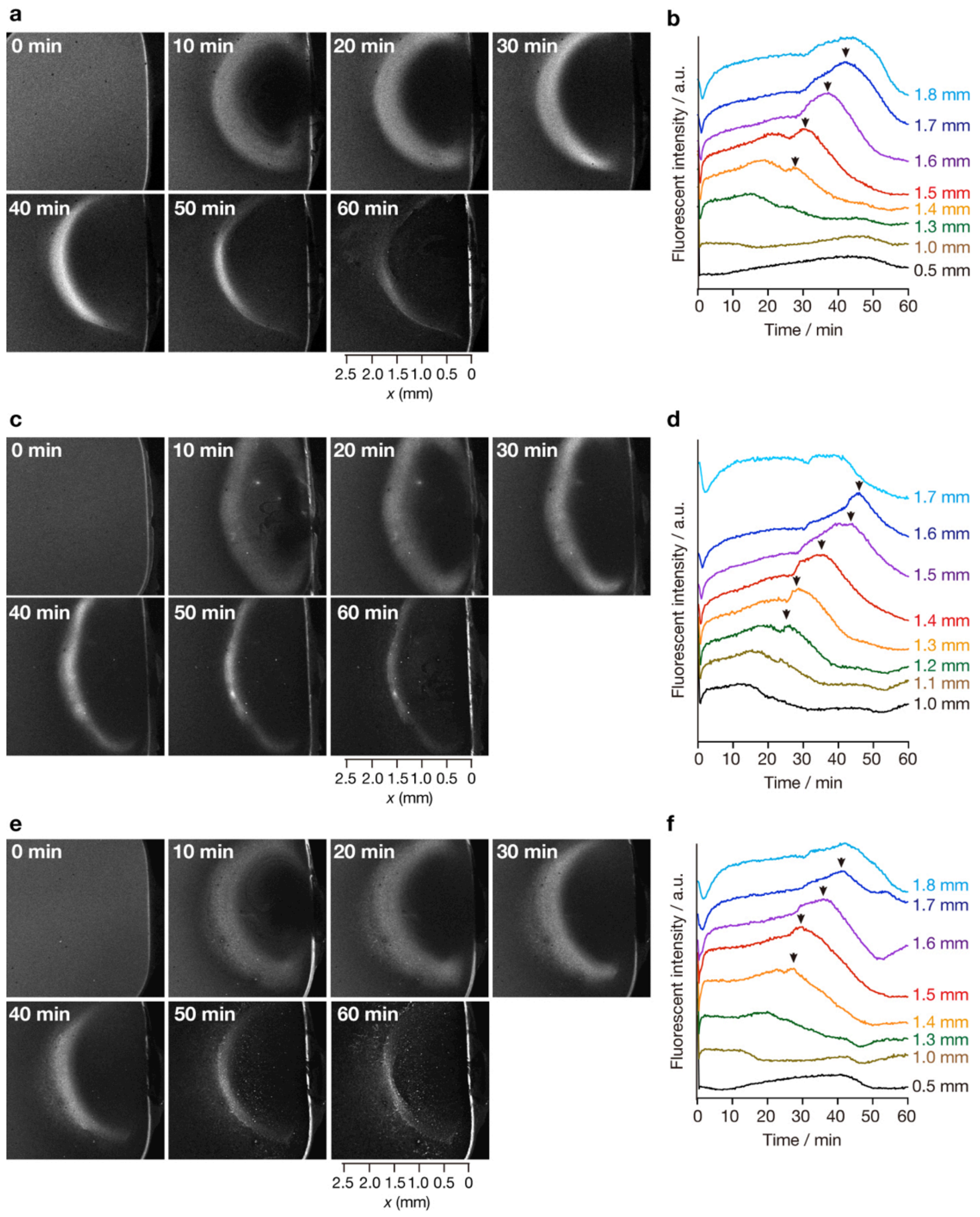

g

Entry	Distance (μm)	Velocity (μm/min)
1	348	21.2
2	348	17.5
3	289	20.8
Average	330 ± 30	20 ± 2

Supplementary Fig. 11 | The velocity of the propagating wave decreased when using a lower amount of GOx. (**a**, **c**, **e**) Time-lapse CLSM imaging of the propagating wave of supramolecular nanofibers. (**b**, **d**, **f**) Time course of fluorescent intensity changes at distinct *x*-coordinates. (**g**) Table of traveling distances and velocity of the propagating wave. The data represent the average ± standard deviation. Condition: [BPmoc-F_3] = 1.6 mM, [BP-TMR] = 0.34 μM, [GOx] = 0.5 mg/mL, [$Zn(NO_3)_2$] = 0.8 mM, [glucose] = 3.2 mM, 50 mM HEPES, pH 7.4, 30 ºC.

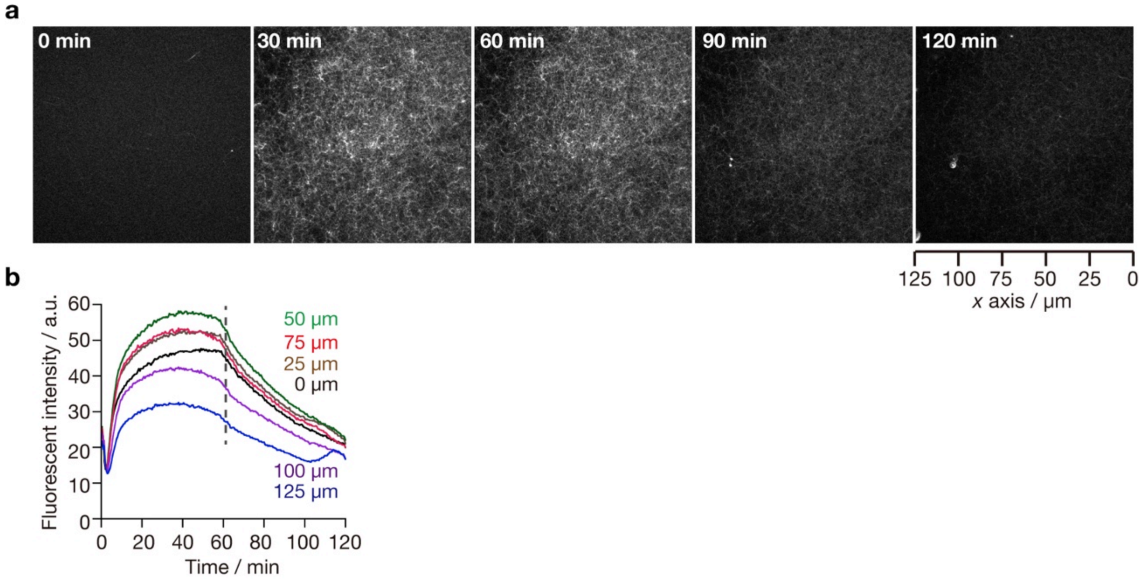

Supplementary Fig. 12 | Propagating wave did not form upon treatment of lower amount of glucose. (**a**) Time-lapse CLSM imaging of homogeneous formation and degradation of BPmoc-F_3 nanofibers upon treatment of $Zn(NO_3)_2$ (0.5 eq) and glucose (1.0 eq). (**b**) Time course of fluorescent intensity changes at distinct *x*-coordinates. Condition: [BPmoc-F_3] = 1.6 mM, [BP-TMR] = 0.34 μM, [GOx] = 1 mg/mL, [$Zn(NO_3)_2$] = 0.8 mM, [glucose] = 1.6 mM, 50 mM HEPES, pH 7.4, 30 ºC.

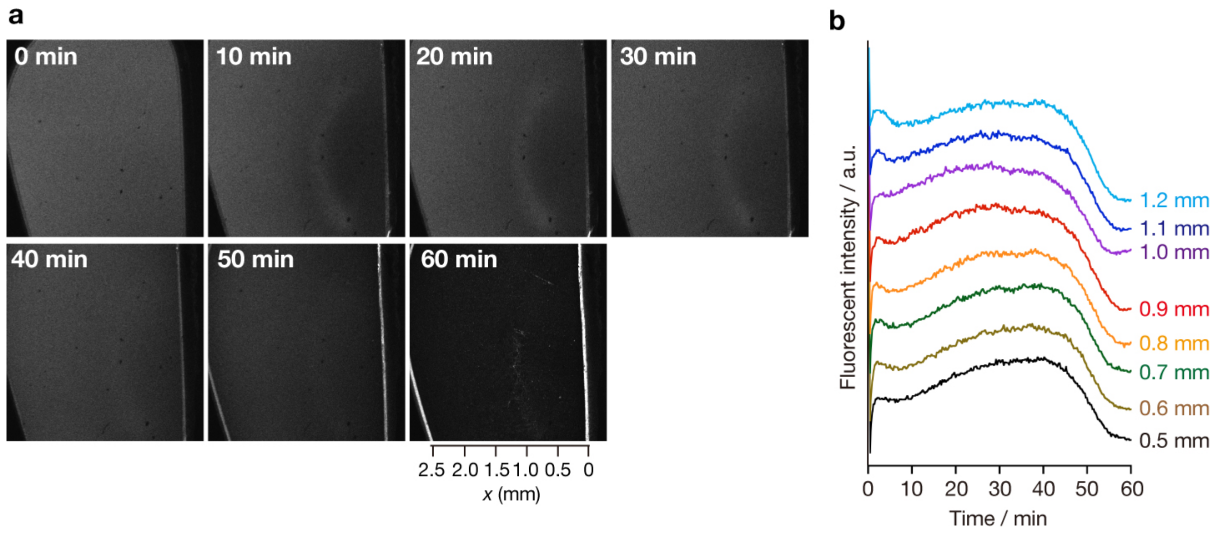

Supplementary Fig. 13 | A propagating wave did not form with a higher amount of GOx. (**a**) Time-lapse CLSM imaging showed that the supramolecular nanofibers hardly formed in the presence of higher amount of GOx. (**b**) Time course of fluorescent intensity changes at distinct *x*-coordinates. Condition: [BPmoc-F_3] = 1.6 mM, [BP-TMR] = 0.34 μM, [GOx] = 2.0 mg/mL, [$Zn(NO_3)_2$] = 0.8 mM, [glucose] = 3.2 mM, 50 mM HEPES, pH 7.4, 30 ºC.

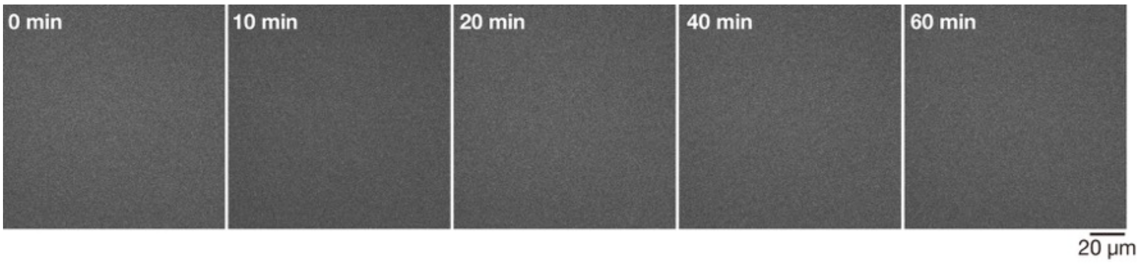

Supplementary Fig. 14 | No fiber formation was observed by simultaneous addition of $Zn(NO_3)_2$ and larger amount of glucose. Time-lapse CLSM imaging of the BPmoc-F_3 droplet by simultaneous addition of $Zn(NO_3)_2$ (0.5 eq) and larger amount of glucose (63 eq). Condition: [BPmoc-F_3] = 1.6 mM, [BP-TMR] = 0.34 μM, [GOx] = 1 mg/mL, [$Zn(NO_3)_2$] = 0.8 mM, [glucose] = 100 mM, 50 mM HEPES, pH 7.4, 30 ºC.

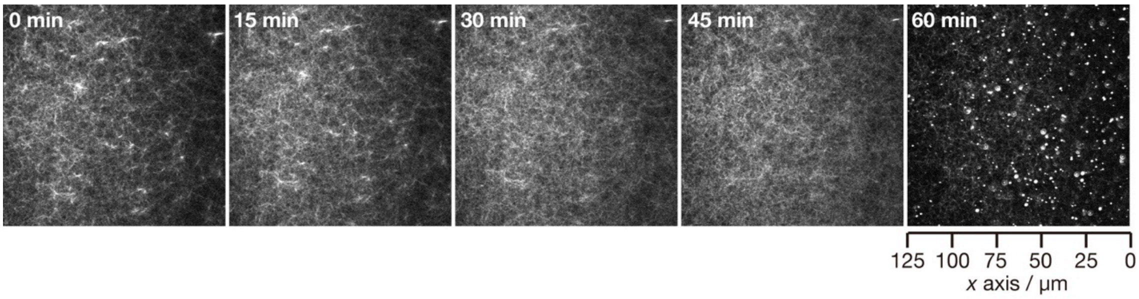

Supplementary Fig. 15 | Sequential treatment of $Zn(NO_3)_2$ followed by glucose. Time-lapse CLSM imaging of degradation of Zn^{2+}-induced BPmoc-F_3 nanofibers upon treatment of glucose. Glucose was added 30 min after $Zn(NO_3)_2$ addition. Time course of fluorescent intensity changes at distinct *x*-coordinates was shown in Fig. 2d. Condition: [BPmoc-F_3] = 1.6 mM, [BP-TMR] = 0.34 µM, [GOx] = 1 mg/mL, [$Zn(NO_3)_2$] = 0.8 mM, [glucose] = 3.2 mM, 50 mM HEPES, pH 7.4, 30 ºC.

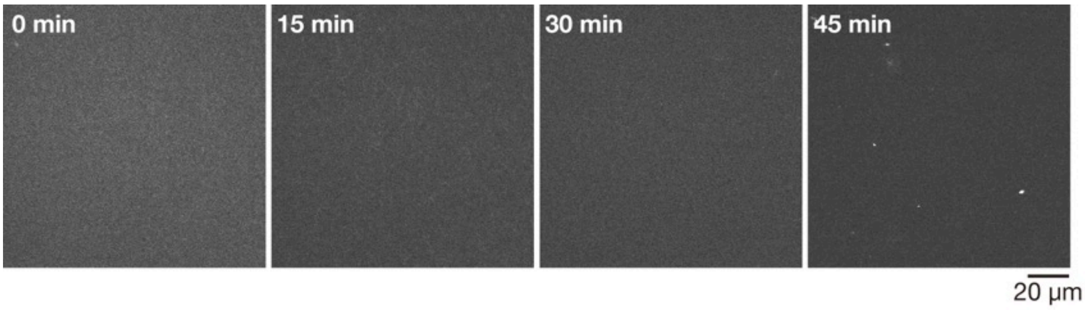

Supplementary Fig. 16 | Sequential treatment of glucose followed by $Zn(NO_3)_2$. Time-lapse CLSM imaging of the droplet of BPmoc-F_3, BP-TMR, and GOx upon addition of glucose followed by $Zn(NO_3)_2$. $Zn(NO_3)_2$ was added 5 min after glucose addition. Condition: [BPmoc-F_3] = 1.6 mM, [BP-TMR] = 0.34 μM, [GOx] = 1 mg/mL, [$Zn(NO_3)_2$] = 0.8 mM, [glucose] = 3.2 mM, 50 mM HEPES, pH 7.4, 30 ºC.

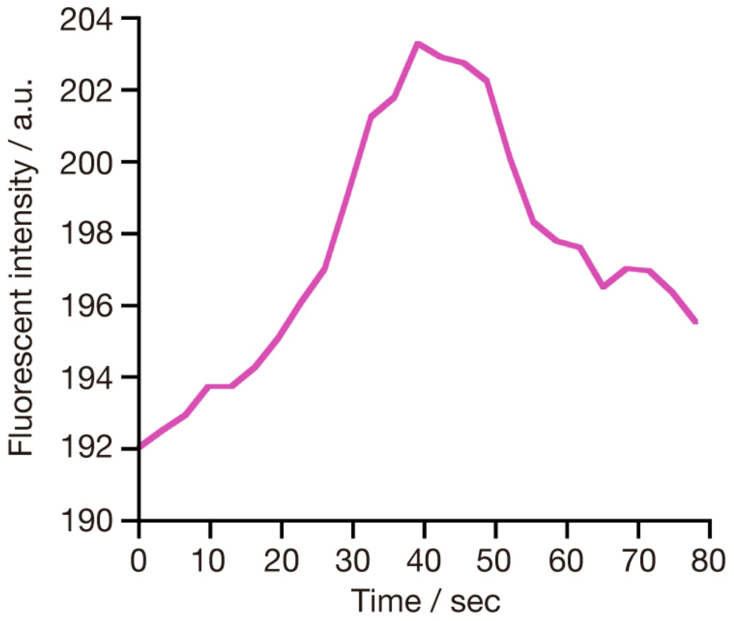

Supplementary Fig. 17 | Time course of fluorescent intensity of BPmoc-F_3 nanofibers. The total fluorescent intensity in the field of view (Fig. 4b) was measured. This time profile indicated the emergence of the propagating wave in the field of view.

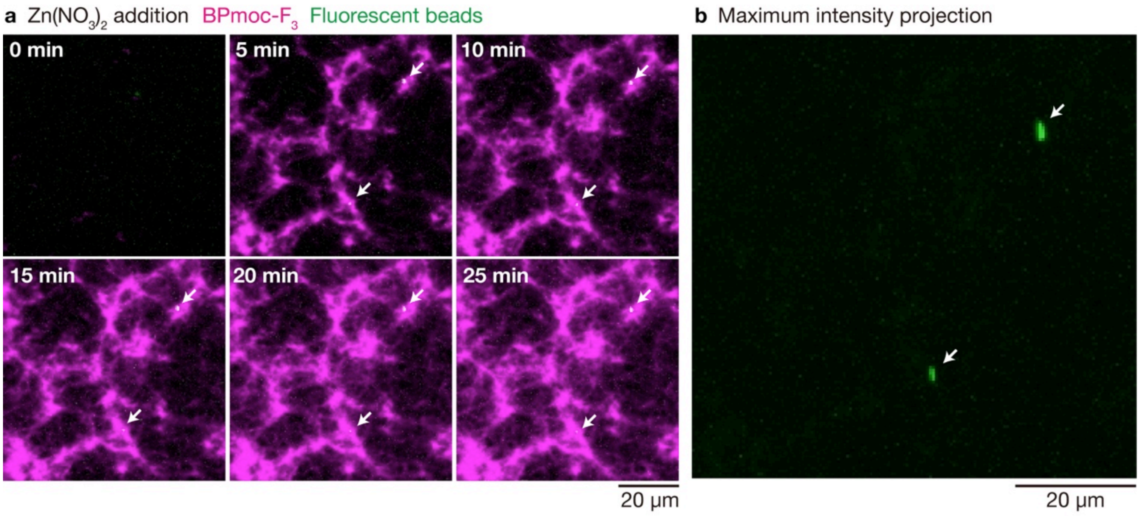

Supplementary Fig. 18 | Time-lapse imaging of the fluorescently-labeled beads under homogeneous nanofiber formation. (**a**) CLSM imaging of the fluorescently-labeled beads under homogeneous nanofiber formation upon addition of $Zn(NO_3)_2$. (**b**) Trajectories of the intensity maxima from the fluorescently labeled beads monitored over 25 min. Under the negative control conditions, the fluorescent-labeled beads did not show any significant displacement during homogenous nanofiber formation and degradation. Condition: [BPmoc-F_3] = 1.6 mM, [BP-TMR] = 0.34 μM, [GOx] = 1.0 mg/mL, [beads] = 20 μg/mL, [$Zn(NO_3)_2$] = 0.8 mM in 50 mM HEPES, pH 7.4, 30 ºC.

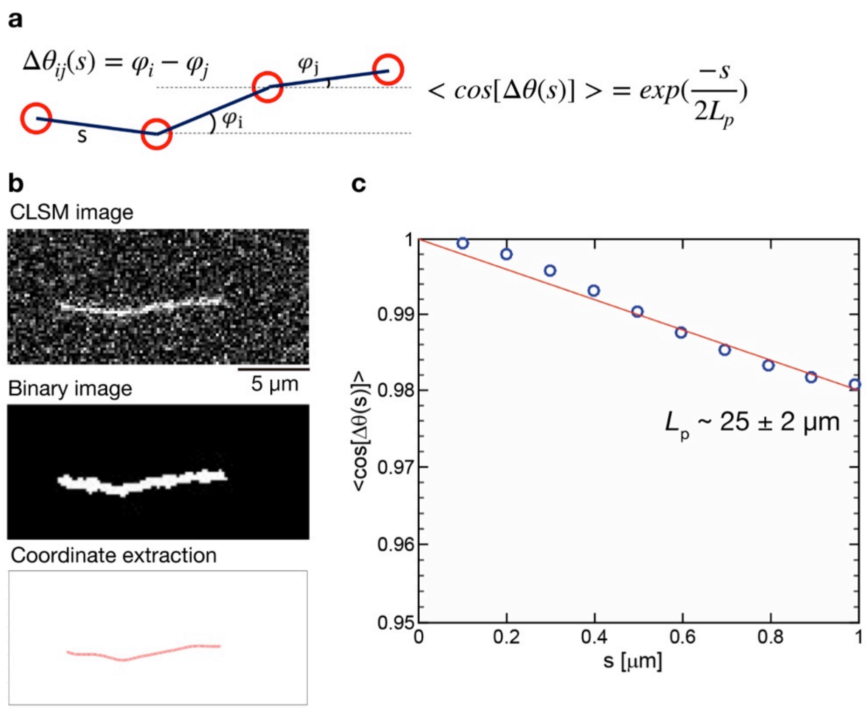

Supplementary Fig. 19 | Determination of the persistence length of a supramolecular nanofiber. (**a**) Mathematical formula of persistence length. (**b**) (top) CLSM, (middle) binary, and (bottom) coordinate extraction images of a supramolecular nanofiber. (**c**) Fitting curve. The persistence length was determined to be *ca.* 25 µm. Condition: [BPmoc-F_3] = 1.2 mM, [BP-TMR] = 0.34 µM, [$Zn(NO_3)_2$] = 0.8 mM in 50 mM HEPES, pH 7.4, 23 ºC.

Supplementary References

1. Schindelin, J. *et al.* Fiji: an open-source platform for biological-image analysis. *Nat. Methods* **9**, 676–682 (2012).
2. Ikeda, M., Tanida, T., Yoshii, T., Kurotani, K., Onogi, S., Urayama, K., Hamachi, I. Installing logic-gate responses to a variety of biological substances in supramolecular hydrogel–enzyme hybrids. *Nat. Chem.* **6**, 511–518 (2014).
3. Onogi, S.; Shigemitsu, H.; Yoshii, T.; Tanida, T.; Ikeda, M.; Kubota, R.; Hamachi, I. *In situ* real-time imaging of self-sorted supramolecular nanofibres. *Nat. Chem.* **8**, 743–752 (2016).
4. Ohta, T.; Kiyose, J.; Mimura. M. Collision of propagating pulses in a reaction-diffusion system. *J. Phys. Soc. Jpn.* **66**, 1551–1558 (1997).
