# Supplementary material for: Force generation by a propagating wave of supramolecular nanofibers
Source: Nat Commun. 2020 Jul 15;11:3541. doi: 10.1038/s41467-020-17394-z (PMC7363860; doi:10.1038/s41467-020-17394-z)
Supplement: Supplementary file 3 — Description of Additional Supplementary Files [file 41467_2020_17394_MOESM3_ESM.pdf]

Description of Additional Supplementary Files

File Name: Supplementary Movie 1
Description: Time-lapse CLSM imaging of BPmoc-F3 nanofiber formation upon treatment of Zn(NO3)2 (shown in Supplementary Fig. 5a). Condition: [BPmoc-F3] = 1.6 mM, [BP-TMR] = 0.34 µM, [Zn(NO3)2] = 0.8 mM, 50 mM HEPES, pH 7.4, 30 ºC. The images were acquired at 2 frame/min. Elapsed time was displayed as hh:mm:ss.

File Name: Supplementary Movie 2
Description: Time-lapse CLSM imaging of degradation of the Zn2+-induced BPmoc-F3 nanofibers upon treatment of glucose (shown in Supplementary Fig. 6a). Condition: [BPmoc-F3] = 1.6 mM, [BP-TMR] = 0.34 µM, [GOx] = 1 mg/mL, [Zn(NO3)2] = 0.8 mM, [glucose] = 3.2 mM, 50 mM HEPES, pH 7.4, 30 ºC. The images were acquired at 2 frame/min. Elapsed time was displayed as hh:mm:ss.

File Name: Supplementary Movie 3
Description: Time-lapse CLSM imaging of the propagating wave of supramolecular nanofibers observed by 100× objectives (shown in Fig. 2b). Condition: [BPmoc-F3] = 1.6 mM, [BP-TMR] = 0.34 µM, [GOx] = 1.0 mg/mL, [Zn(NO3)2] = 0.8 mM, [glucose] = 3.2 mM in 50 mM HEPES, pH 7.4, 30 ºC. The images were acquired at 2 frame/min. Elapsed time was displayed as mm:ss.

File Name: Supplementary Movie 4
Description: Time-lapse CLSM imaging of the propagating wave of supramolecular nanofibers observed by 4× objectives (shown in Fig. 2e). Condition: [BPmoc-F3] = 1.6 mM, [BP-TMR] = 0.34 µM, [GOx] = 1.0 mg/mL, [Zn(NO3)2] = 0.8 mM, [glucose] = 3.2 mM in 50 mM HEPES, pH 7.4, 30 ºC. The images were acquired at 4 frame/min. Elapsed time was displayed as mm:ss.

File Name: Supplementary Movie 5
Description: Time-lapse CLSM imaging of the propagating wave of supramolecular nanofibers observed by 4× objectives with a lower amount of GOx (shown in Supplementary Fig. 11a). Condition: [BPmoc-F3] = 1.6 mM, [BP-TMR] = 0.34 µM, [GOx] = 0.5 mg/mL, [Zn(NO3)2] = 0.8 mM, [glucose] = 3.2 mM in 50 mM HEPES, pH 7.4, 30 ºC. The images were acquired at 4 frame/min. Elapsed time was displayed as hh:mm:ss.

File Name: Supplementary Movie 6
Description: Time-lapse CLSM imaging of homogeneous formation and degradation of BPmoc-F3 nanofibers upon treatment of Zn(NO3)2 and a lower amount of glucose (shown in Supplementary Fig. 12a). Condition: [BPmoc-F3] = 1.6 mM, [BP-TMR] = 0.34 µM, [GOx] = 1 mg/mL, [Zn(NO3)2] = 0.8 mM, [glucose] = 1.6 mM, 50 mM HEPES, pH 7.4, 30 ºC. The images were acquired at 2 frame/min. Elapsed time was displayed as hh:mm:ss.

File Name: Supplementary Movie 7
Description: Time-lapse CLSM imaging of degradation of the Zn2+-induced BPmoc-F3 nanofibers upon treatment of glucose (shown in Supplementary Fig. 15). Glucose was added 30 min after Zn(NO3)2 addition. Condition: [BPmoc-F3] = 1.6 mM, [BP-TMR] = 0.34 µM, [GOx] = 1 mg/mL, [Zn(NO3)2] = 0.8 mM, [glucose] = 3.2 mM, 50 mM HEPES, pH 7.4, 30 ºC. The images were acquired at 2 frame/min. Elapsed time was displayed as hh:mm:ss.

File Name: Supplementary Movie 8
Description: Numerical simulation of time dependent concentration changes of (upper left) supramolecular nanofibers, (upper right) monomer, (lower left) formation, and (lower right) degradation stimuli (shown in Fig. 3a).

File Name: Supplementary Movie 9
Description: CLSM imaging of the displacement of the fluorescently-labeled beads under the propagating wave (shown in Fig. 4b). Condition: [BPmoc-F3] = 1.6 mM, [BP-TMR] = 0.34 µM, [GOx] = 1.0 mg/mL, [beads] = 20 µg/mL, [Zn(NO3)2] = 0.8 mM, [glucose] = 3.2 mM in 50 mM HEPES, pH 7.4, 30 ºC. The images were acquired at 18 frame/min. Elapsed time was displayed as sec.

File Name: Supplementary Movie 10
Description: CLSM imaging of the fluorescently-labeled beads under homogeneous nanofiber formation (shown in Supplementary Fig. 18a). Condition: [BPmoc-F3] = 1.6 mM, [BP-TMR] = 0.34 µM, [GOx] = 1.0 mg/mL, [beads] = 20 µg/mL, [Zn(NO3)2] = 0.8 mM in 50 mM HEPES, pH 7.4, 30 ºC. The images were acquired at 18 frame/min. Elapsed time was displayed as mm:ss.

File Name: Supplementary Movie 11
Description: CLSM imaging of the fluorescently-labeled beads under homogeneous nanofiber degradation (shown in Fig. 4d). Glucose was added 1 h after Zn(NO3)2 addition. Condition: [BPmoc-F3] = 1.6 mM, [BP-TMR] = 0.34 µM, [GOx] = 4 1.0 mg/mL, [beads] = 20 µg/mL, [Zn(NO3)2] = 0.8 mM, [glucose] = 3.2 mM in 50 mM HEPES, pH 7.4, 30 ºC. The images were acquired at 18 frame/min. Elapsed time was displayed as mm:ss.

File Name: Supplementary Movie 12
Description: Thermal fluctuation of a supramolecular fiber (shown in Supplementary Fig. 19). Condition: [BPmoc-F3] = 1.2 mM, [BP-TMR] = 0.34 µM, [Zn(NO3)2] = 0.8 mM in 50 mM HEPES, pH 7.4, 23 ºC. The images were acquired at 33 frame/sec. Elapsed time was displayed as sec.
